# Aggressive Adenoid Cystic Carcinoma of Maxillary Sinus in a 43-Year-Old Male: Rare Case Report and Review of Literature

**DOI:** 10.1155/2017/2324717

**Published:** 2017-09-28

**Authors:** Koorosh Rahmani, Shokouh Taghipour Zahir, Mohammad Baghi Yazdi, Alireza Navabazam

**Affiliations:** ^1^Shahid Sadoughi University of Medical Sciences, Yazd, Iran; ^2^Student Research Committee, Shahid Sadoughi University of Medical Sciences, Yazd, Iran

## Abstract

Adenoid cystic carcinoma (ACC) is a rare malignant tumor, mostly involving the minor salivary glands. Herein, we present a case of ACC in a 43-year-old man with symptoms of dental abscess as the initial presentation of the tumor. In spiral computed tomography (CT) scan, soft tissue mass with the erosion of maxillary sinus wall on the right side of the alveolar ridge was evident. Histopathological examination of the excised tumor with immunohistochemical studies (C-kit, Vimentin, pan-cytokeratin, p53, p63, and ki67 positive reaction) confirmed grade 2 ACC in the maxillary sinus. The patient underwent hemimaxillectomy and right-neck dissection. Due to the extension of tumor cells excessively into the surrounding tissues and involvement of orbital bone, complete and total resection of the tumor with safe margins could not be done. After surgery, adjuvant radiotherapy was considered for the patient. At the end of treatment, the patient lost his eye vision. Seventeen months from initial diagnosis, he was still alive without lung or distant metastasis.

## 1. Introduction

ACC is a rare malignant tumor with an incidence of 1% of all malignant neoplasms of the oral cavity and maxillofacial area [1]. It mostly affects the minor salivary glands and is histologically composed of small basaloid cells arranged in solid, cribriform, and trabecular pattern [1, 2]. Regardless of the benign histopathological appearance of ACC, it potentially has local invasion with late recurrence rate and distant metastasis [3]. The most prognostic predictive factors are tumor size, grade, stage, lymph node involvement, neural invasion, and margin status. The high grade ACC (the especially solid type of ACC) can be misdiagnosed with other malignant neoplasms such as basaloid squamous cell carcinoma, neuroendocrine tumors, and adenosquamous cell carcinoma, and immunohistochemistry is a reliable approach used to differentiate such a salivary gland tumor from others [4, 5]. Herein, we report a case of ACC with an uncommon clinical presentation such as tooth abscess, infectious status, and early involvement of regional lymph nodes that extended into the surrounding tissues with orbital bone involvement and which was misdiagnosed first as basaloid squamous cell carcinoma.

## 2. Case Presentation

**Figure 1:**
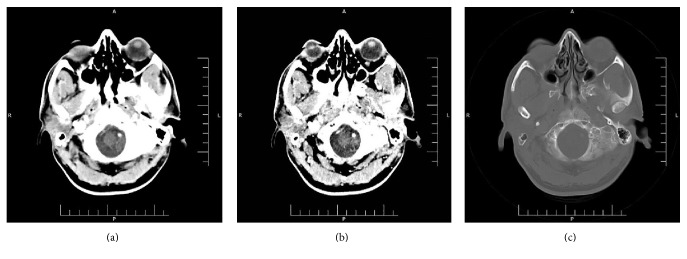
(a) Tri-dimensional CT scan reveals increasing of the thickness of right maxillary sinus soft tissue. (b) Massive destruction of right maxillary body. (c) Right maxillary sinus opacity with destruction of sinus wall.

**Figure 2:**
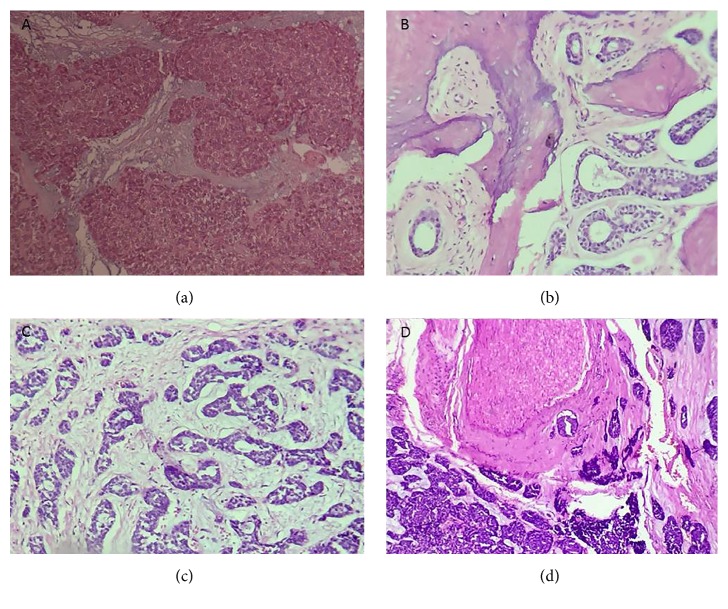
(a) Nests of neoplastic cells with peripheral palisading pattern within fibromyxoid stroma (Hematoxylin and eosin staining, original magnification ×20). (b) Marrow involvement of orbital and maxillary sinus bones. (c) Neoplastic cells have dark hyperchromatic nuclei arranged in cribriform, solid nests, and trabecular formations. (d) Perineural invasion in the depth of tumor is evident (Hematoxylin and eosin stain, original magnification ×20).

**Figure 3:**
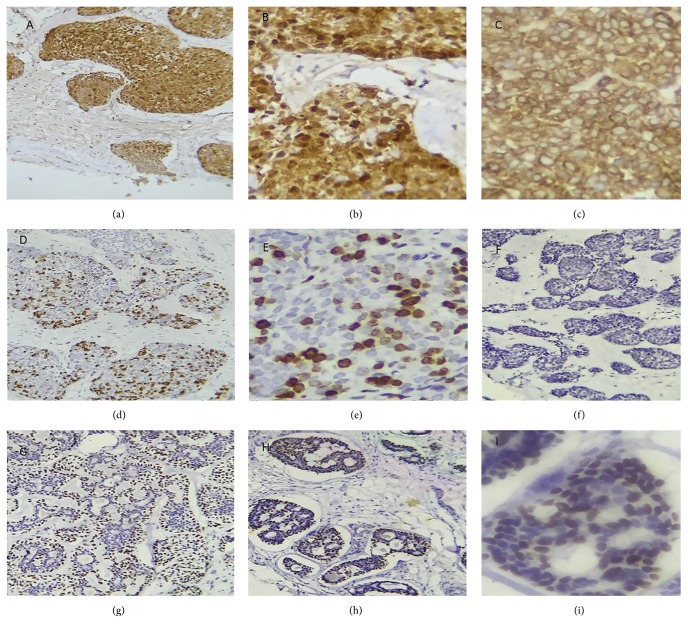
(a), (b) Neoplastic cells have a positive reaction pattern for S-100 (immunohistochemical staining, original magnification, ×20 and ×40). (c) Positive reaction pattern for pan-cytokeratin (IHC staining original magnification ×40). (d), (e) Positive reaction pattern for Ki67 (IHC staining, ×20 and ×40). (f) Negative reaction pattern of neoplastic cells for p63 in initial biopsy specimen (IHC staining, ×20). (g), (h), (i) Positive reaction pattern of neoplastic cells for p63 in secondary excised lesion (immunohistochemical staining, ×20 and ×40).

In March 2016, a 43-year-old male was referred to an odontology clinic with toothache and fever. His previous medical history was unremarkable. On physical examination, the upper airway was open but the right nasal cavity appeared to be closed by tumoral lesion. Edema of the right palate with the involvement of the hard and soft palates was seen. In the right side of the neck, multiple lymph nodes were touched along the sternocleidomastoid (SCM). Owing to high grade fever, complete laboratory blood tests were requested. In complete blood cell count (CBC), increase of white blood cells 11,500 per microliter (3.5–10) was detected. It appeared that the patient had a source of infection in the mouth. Despite receiving high doses of antibiotics, no signs of healing were observed. Also, it seemed that the main lesion was beyond simple infection. Ultrasound investigation of the right lateral neck chain revealed two heteroechoic lymph nodes with irregular lobulated borders containing internal necrosis, with one of them measuring 21 × 14 mm (with conglomerate feature composed of edema associated with matting), and the other one measuring 12 × 24 mm (with the suspicious feature of malignancy) and containing necrosis. Another lymph node was situated anteriorly to the carotid artery with a reactive feature measuring 9 × 3 mm in diameter. Left neck ultrasound revealed multiple lymph nodes with some of them having a reactive feature and one having a suspicious feature for malignancy in the left supraclavicular area. Tri-dimensional head and neck computed tomography (CT) scan revealed increasing thickness of soft tissue of the right maxillary sinus area with massive destruction of the right maxillary sinus wall (Figures 1(a), 1(b), and 1(c)). The patient underwent biopsy via endoscopic procedure of right maxillary sinus mass in an outpatient maxillofacial surgery clinic. The first pathology report was basaloid squamous cell carcinoma, without any immunohistochemical study. For confirmation of diagnosis and to decide treatment protocol, the sample was sent back for consultation to our center. Histopathological examination of Hematoxylin and Eosin (H&E) stained glass slides revealed a tumoral lesion composed of dark hyperchromatic neoplastic cells with round nuclei (basaloid-like cells) that were arranged in solid nests and acinar structures with peripheral palisading pattern in the fibromyxoid stroma without characteristic features of adenoid cystic carcinoma (Figure 2(a)). In differential diagnosis, solid type of ACC was considered. For definite diagnosis IHC studies were done and neoplastic cells showed positive reaction pattern to the S-100 (diffusely cytoplasmic and nuclear staining) (Figures 3(a) and 3(b)), pan-cytokeratin (AE1/AE3) (cell membrane staining) (Figure 3(c)), Vimentin, p53, C-kit, alpha smooth muscle actin, and also a ki67 labeling index of 60% (nuclear staining) (Figures 3(d) and 3(e)) (Dako, Denmark). All neoplastic cells showed negative staining pattern for p63 (Figure 3(f)). The negative reaction of the malignant cells for p63 were not justifiable, because each of the two tumors under discussion (adenoid cystic carcinoma and basaloid squamous cell carcinoma) should have a positive reaction to this marker, and only their pattern of staining was different. Therefore, according to histopathologic findings, this tumor was suggested to be basaloid squamous cell carcinoma. The patient underwent hemimaxillectomy, semi-hard palate resection, cheek bone, right neck, submental and submandibular lymph nodes dissection and resection of the fascia of maxillary sinus, nose, and soft palate, and also palatine tonsillectomy. Due to the excessive extension of tumor cells into the surrounding tissues and the involvement of orbital bone, safe margins could not be made during the surgery (Figure 2(b)). The histopathological examination of received tissues showed tumoral lesion composed of small, dark, round cells with angulated nuclei, arranged in solid nests, trabeculae, cribriform, and tubular structures within fibromyxoid stroma in the depth of the tumor, with characteristic features of ACC tumors (Figure 2(c)). Numerous perineural and intraneural invasions with lymph node metastasis were present (Figure 2(d)). IHC studies revealed compartment staining pattern of neoplastic cells for p63 (Figures 3(g), 3(h), and 3(i)). Based on histopathological and IHC findings, the final diagnosis of tumor was grade 2 ACC. After surgery, adjuvant radiotherapy was considered for the patient. The patient has taken three courses of chemotherapy and thirty courses of radiotherapy. At the end of radiation therapy, he lost his right eye vision.

Follow-up: seventeen months from initial diagnosis, he was still alive without lung or distant metastasis.

## 3. Discussion

ACC is a rare malignant neoplasm of salivary glands of the head and neck region. However, although major and minor salivary glands are affected by this tumor, 50%–70% of cases are presented in minor salivary glands [1, 2]. ACC affects both males and females, without any gender predilection, especially in their fifth to seventh decades of life [3]. Although a study by Gill and Frattali, 2015, reported slight female predominance, Gondivkar et al., 2011, mentioned that there was no gender predominance [5, 6]. Based on tumor location, patients have variable clinical symptoms and signs, but pain is a common clinical finding due to an early perineural invasion of neoplastic cells [6–8]. ACC grows slowly over time and most patients presented advanced stages [8]. The carcinoma cells originating from duct-type epithelial and myoepithelial cells of salivary glands have a dark blue nucleus, arranged in solid nests, tubular, cribriform patterns with pseudo-cystic spaces [3, 4]. The tumor is graded by the pattern of neoplastic cells arrangement [5, 6]. Solid type of ACC is composed of dark neoplastic cells arranged in a solid nests pattern in more than 30% of the entire tumor and has an aggressive behavior with high recurrence rate and lymph nodes metastasis, and most patients present at an advanced stage [3, 4]. In the differential diagnosis of ACC, polymorphous low-grade adenocarcinoma, basaloid squamous cell carcinoma, small cell neuroendocrine carcinoma, and adenosquamous cell carcinoma can be considered. In BSSC, neoplastic cells have dark small irregular nuclei arranged in solid nests with peripheral palisading pattern and are centered by keratinized or non-keratinized squamous cells [1, 2]. The stroma of BSSC is highly collagenized. However, neoplastic cells in ACC arranged in cribriform, solid, or tubular pattern with perineural invasions [4, 6]. Also, in ACC neoplastic cells do not have squamous differentiation and small myoepithelial cells which have a dark angular nucleus surround pseudoglandular spaces [6]. In dedifferentiated solid type (grade 2, Van Weert 2015) of ACC anaplastic cells have an abundant cytoplasm arranged in islands within desmoplastic stroma that simulate basaloid squamous cell carcinoma and IHC staining helps to differentiate this type of ACC from BSSC [5, 9]. ACC cells have a positive reaction pattern for Vimentin, pan-cytokeratin, C-kit, p53, Ki67, and also alpha smooth muscle actin in comparison with neoplastic cells in BSSC that have a negative reactive pattern for smooth muscle actin [3–5]. ACC cells reveal compartment staining pattern for p63 and also a strongly positive reactive pattern for Vimentin compared with BSCC [8]. In BSCC, neoplastic cells show diffuse positive reaction pattern to p63; however, ACC cells have a compartment positive reaction pattern for p63, which means that A) positive peripheral cells surround centered cells with negative reactive pattern or B) negative-stained peripherally located cells are centered by neoplastic cells which show positive reaction pattern for p63 [9]. In our case, all neoplastic cells showed negative reaction pattern for p63 in the first biopsy specimen, which was the cause of the misdiagnosis of BSCC, and, also, the negative reaction pattern was inconsistent with other literature reviews for diagnosis of ACC [10]. Terada 2013 reported that neoplastic cells in oral ACC had a negative staining pattern for S-100 but, in contrast to their findings, in our case, neoplastic cells had a strongly positive reaction pattern for S-100 protein [7]. Although adenoid cystic carcinoma cells stained positive for p63, it seems that in solid and high grade tumors neoplastic cells in solid areas could not be stained by p63 antibody and negative staining pattern should not mislead to diagnosis of other neoplastic lesions. Small cell neuroendocrine carcinoma cells react positively for neuroendocrine markers such as NSE, synaptophysin, and chromogranin A, despite ACC cells which have negative staining pattern for these markers [4, 5]. Lymph node involvement or distant metastases occur many years after first tumor presentation [7, 8]. The behavior of solid type of ACC (grade 2) is different from grade 1 tumors and presents in more advanced stages in initial presentation, as was the case in our patient [7]. Although lung metastasis has been reported more than regional lymph nodes involvement, in our case lung metastasis was not present, although regional lymph nodes were involved [5–8]. The main treatment protocol for aggressive ACC is surgery with free surgical margins followed by radiotherapy and, in advanced cases, adjuvant radiotherapy is advised [1, 2, 6]. In our case, due to orbital bone involvement as well as the immense extent of the tumor, surgery was not completed and we did not have safe surgical margins. Adjuvant radiotherapy was considered for the patient. At the end of radiation therapy, he lost his right eye vision.

## 4. Conclusions

Adenoid cystic carcinoma is a malignant tumor with indolent growth pattern. For differentiation of the solid type of ACC, IHC studies are necessary. After a long time of occurrence, local recurrence or distant metastasis to other places such as lungs and liver is seen. Unlike the classical cribriform type that has a slow growth, mixed and solid types have aggressive behavior and can spread to surrounding tissues and may even involve regional lymph nodes or distant metastasis at the time of discovery. Overall, there is no cure for this type of tumor.
